# Pulse-response measurement of frequency-resolved water dynamics on a hydrophilic surface using a Q-damped atomic force microscopy cantilever

**DOI:** 10.3762/bjnano.3.29

**Published:** 2012-03-19

**Authors:** Masami Kageshima

**Affiliations:** 1 Department of Physics, Tokyo Gakugei University, 4-1-1 Nukui-kita-machi, Koganei, Tokyo 184-8501, Japan

**Keywords:** atomic force microscopy, hydration, pulse-response, quality-factor control, viscoelasticity

## Abstract

The frequency-resolved viscoelasticity of a hydration layer on a mica surface was studied by pulse-response measurement of a magnetically driven atomic force microscopy cantilever. Resonant ringing of the cantilever due to its 1st and 2nd resonance modes was suppressed by means of the Q-control technique. The Fourier–Laplace transform of the deflection signal of the cantilever gave the frequency-resolved complex compliance of the cantilever–sample system. The significant viscoelasticity spectrum of the hydration layer was successfully derived in a frequency range below 100 kHz by comparison of data obtained at a distance of 300 nm from the substrate with those taken in the proximity of the substrate. A positive value of the real part of the stiffness was determined and is attributed to the reported solidification of the hydration layers.

## Introduction

Liquid solvation is a phenomenon common to a large variety of liquid–solid interfaces [1]. In particular, water solvation, or hydration, on hydrophilic surfaces has drawn interest because of its relevance to biological phenomena on the molecular scale. The dynamical properties of hydrated water have been reported to largely differ from those of bulk water based on the analysis of results from various macroscopic experimental approaches [2–4]. Among the new experimental methods developed in the last few decades, atomic force microscopy (AFM), which was originally invented as an imaging method, has also manifested its potential as a site-specific profiling tool of force interaction. Utilizing the high spatial resolution of AFM, various intriguing properties of liquid solvation [5–19], especially hydration [8–9,11–15,18–19], have been newly revealed. It should be noted that, in addition to its high spatial resolution, AFM possesses a distinguished aptitude for dynamical measurements, which is mainly due to the cantilever sensor having the character of a well-defined oscillator. Measurement of the complex response function to oscillatory stress of the sample under study, i.e., the viscoelasticity, is a common approach for studying the dynamical properties of matter, especially so-called soft matter.

It is remarkable that the time-scales of hydration dynamics reported, based on AFM [11,13,15,19] and other mechanical measurements [20–21], differ by orders of magnitude from even those reported from conventional macroscopic hydration measurements, and thus are enormously longer than the bulk value of about 8 ps derived from dielectric relaxation measurements. Here it should be noted that the dielectric measurements detect rotational relaxation of the molecules and therefore should not necessarily coincide with mechanical measurements, such as AFM, in which the translational motion of molecules is considered to play a role. Microscopic viscoelasticity measurements using the AFM hold the potential to approach water hydration from an aspect that has been little explored. At the moment viscoelasticity measurements of hydrated water using the AFM have mostly been carried out only at a single frequency. As a matter of course, interest is oriented toward the mapping of the frequency-resolved viscoelasticity spectrum. The number of reports of the frequency-resolved viscoelasticity analysis of soft matter using the AFM is quite limited [22–25].

Using the method of exciting the AFM cantilever with a well-characterized magnetic force [26–27], attempts have been made to measure the frequency-resolved viscoelasticity spectrum of soft-matter systems. The most straightforward approach is a frequency-domain measurement, in which an oscillatory stress is applied to the cantilever interacting with the sample while its frequency is swept, and the viscoelasticity of the sample is derived from the transfer function of the cantilever response. This was applied to a single polymer chain tethered between the probe and the substrate [24]. Another approach contrasting with the frequency-domain measurement is a time-domain measurement in which the time-dependent response to a stress pulse or step is analyzed [28]. Implementation of a simple step-response measurement based on AFM was exemplified previously [25]. In this measurement a step stress is applied to the cantilever and the response signal *u*(*t*) is converted to the corresponding frequency response function, i.e., a complex compliance 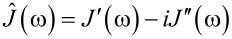, by Fourier–Laplace transformation as,

[1]
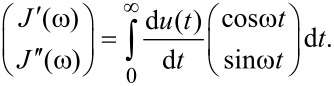


Actually, the time differentiation of *u*(*t*) required prior to Fourier–Laplace transformation is disadvantageous with respect to the signal-to-noise ratio. In the previous report of the step-response measurement the inferior signal-to-noise ratio of the signal hindered detailed analysis. In the present report a pulse-response measurement in which differentiation of the response signal can be dispensed with is described. The viscoelastic response of water in the proximity of hydrophilic mica surface is extracted as a frequency spectrum.

## Results and Discussion

**Figure 1:**
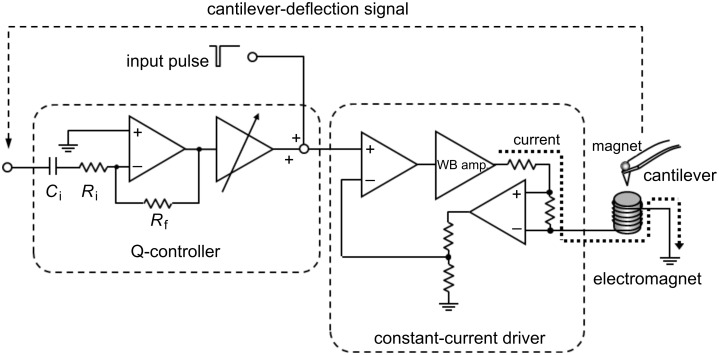
Schematic diagram of cantilever regulation by means of magnetic force. The system consists of a Q-control circuit with a differentiator and a wide-band constant-current driver.

**Figure 2:**
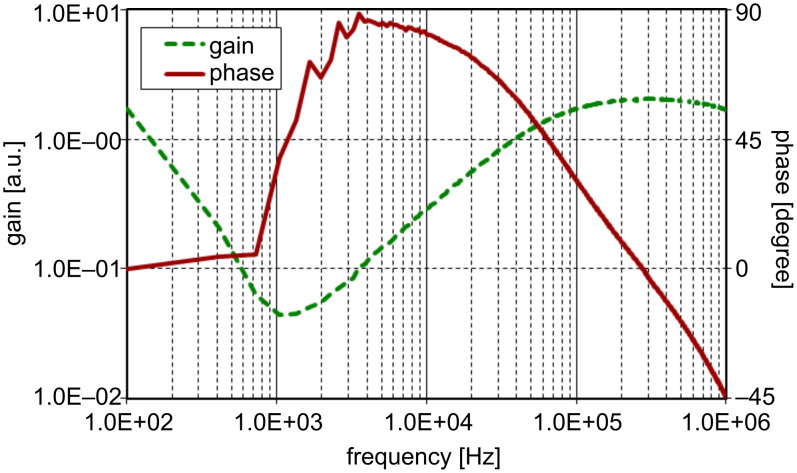
Transfer function of the differentiator in the Q-control circuit with a cutoff frequency of about 80 kHz. The solid and broken lines indicate phase and gain, respectively.

The experimental setup for the magnetic control of the cantilever is essentially similar to the one reported previously [25] and is briefly described below. Since in the present approach the viscoelastic response of the composite system of the cantilever and sample is measured, if the response of the cantilever is too strong it can screen the contribution of the sample. In order to suppress the resonant response of the cantilever the technique of quality-factor-control (Q-control) [29] is employed. The device for magnetic driving of the cantilever consists of two sections; a Q-control circuit for suppression of resonant ringing and a wide-band electromagnet driver, as shown in Figure 1. The Q-control section has an op-amp differentiator, for conversion of the cantilever deflection into velocity, and an amplifier. Although it is ideal to suppress multiple resonance modes independently, the implementation is not realistic; independent setting of feedback gains requires filters in the circuit, which would inevitably perturb its phase response. It is much more practical to cover multiple resonance modes with a single differentiator having a fairly large bandwidth. Since the gain of a differentiator is proportional to the frequency, a cutoff frequency *f*_c_* =* (2π*R*_i_*C*_i_)^−1^ is set to ca. 80 kHz. Figure 2 shows the gain and phase of the Q-control section measured with its pulse input shunted to the ground and the amplifier gain set to the typical operational condition. It is well known that an ideal differentiator has a gain proportional to the frequency and a phase at 90 degrees to that of the input. In Figure 2, the differentiated signal can be detected above the noise at around 1 kHz and the phase reaches 90 degrees at around 4 kHz. The influence of the cutoff, however, soon starts to make it deviate from 90 degrees as the frequency increases. Since a phase error of 45 degrees is often used as a standard for secure feedback, the operation range of this Q-control circuit is evaluated to be from 1.2 to 60 kHz, over which also the gain is almost linear with the frequency. As an inevitable result of using the differentiator, the gain of the Q-control feedback increases with the frequency. This is, however, advantageous in compensating the increase in effective stiffness with mode number [30]. The subsequent electromagnet-driver section is a constant-current amplifier that detects the load current through a resistor inserted in series with the load and keeps it proportional to the input voltage signal with the help of a wideband amplifier inserted in the feedback loop. The net bandwidth of the constant-current driver and the electromagnet is larger than 1 MHz [24–25], which is sufficient for the present measurement.

**Figure 3:**
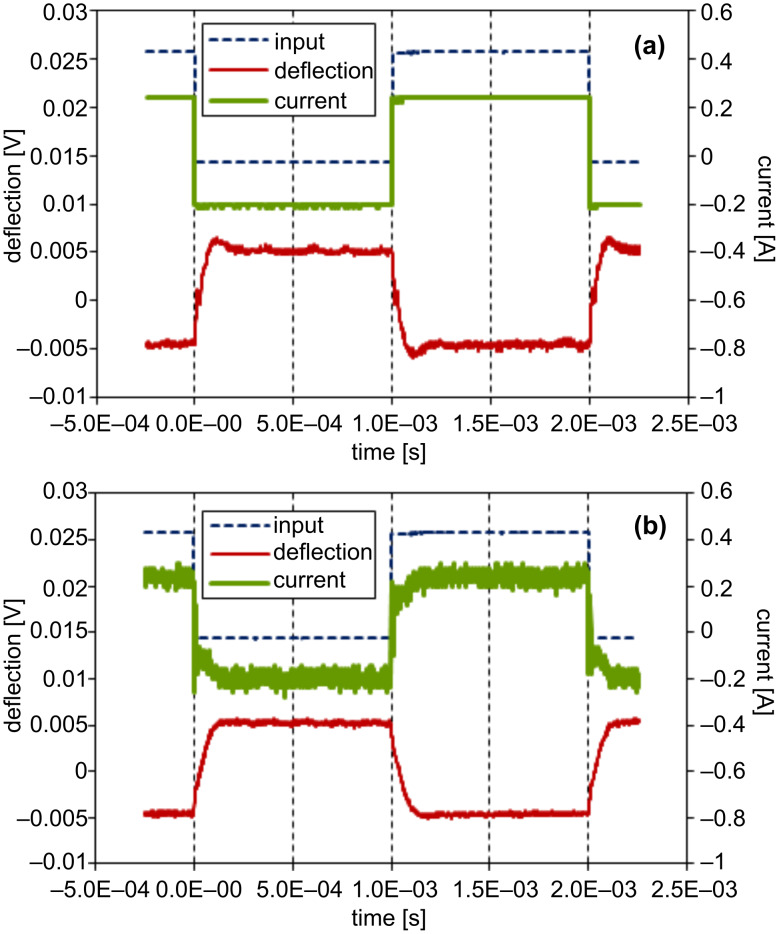
Effect of Q-control on the step-response of the cantilever recorded at about 700 nm from a mica substrate in water with zero Q-control gain (a) and with the optimum gain (b). The fine broken, thick solid, and fine solid lines indicate the input-voltage signal, current in the electromagnet, and cantilever deflection, respectively. The cantilever swing amplitude corresponds to about 4 nm.

**Figure 4:**
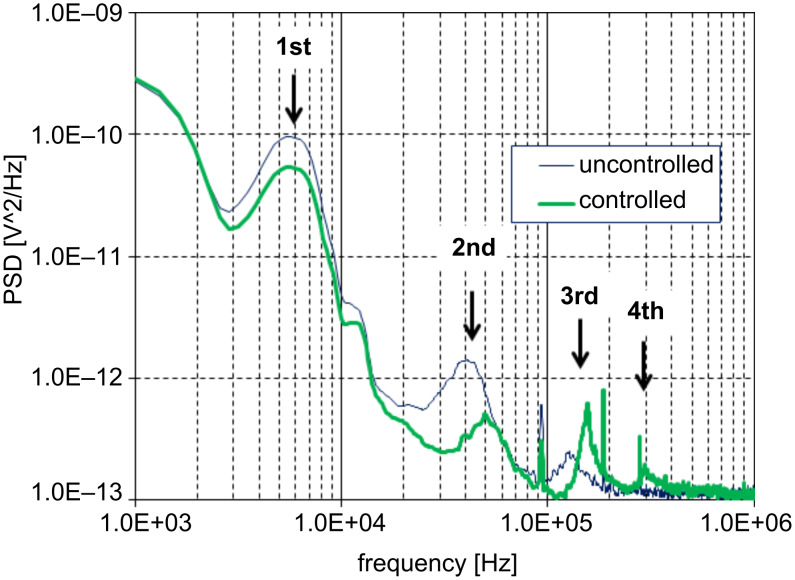
Comparison of the power spectrum density (PSD) of thermal noise in the cantilever-deflection signal before (fine line) and after (thick line) Q-control gain optimization. The peaks in the noise density at the 1st and 2nd resonance modes are suppressed whereas the peaks at the 3rd and 4th modes are enhanced due to the cutoff frequency of 80 kHz.

The measurement was carried out with a 0.03 N/m silicon nitride cantilever integrated with a probe tip. The tip surface was cleaned by UV irradiation in air prior to the installation. After the tip was brought into contact with a freshly cleaved mica substrate, the sample stage of the AFM apparatus was readjusted to give an appropriate tip–substrate separation. For the reduction of noise, the wave data were averaged 256 times. Prior to the measurement a 500 Hz square wave with a duty cycle of 50% was applied to the driving circuit for Q-control gain adjustment. Figure 3a shows the input wave, the current in the electromagnet, and the cantilever deflection recorded with a Q-control gain of zero at a tip–substrate separation of ca. 700 nm. The cantilever deflection shows undulation due to the 1st mode resonance and also a spikelike shoulder due to the 2nd mode. With the Q-controller gain adjusted, the feedback action is superimposed on the current and the ringing features in the deflection signal disappear as shown in Figure 3b. Although the profile of the actual coil current is no longer identical to the input signal due to the Q-control feedback as shown in Figure 3b, a simple consideration of the transfer function reveals that the situation is effectively identical to the case of driving a virtual, resonance-free cantilever with the input waveform [25]. The Q-control gain was fixed to this value throughout the measurement. Figure 4 shows a comparison of the power spectrum density (PSD) of thermal noise in the cantilever-deflection signal before and after Q-control gain optimization. The peaks in the noise density at the 1st and 2nd resonance modes are suppressed, whereas the peaks at the 3rd and 4th modes are enhanced as a result of the above-mentioned cutoff frequency, although this is not harmful for the measurement.

It is crucial to accurately regulate the tip–substrate distance for a reliable measurement. In the case of the present pulse-response measurement, however, an appropriate signal for feedback regulation is not present. As a compromise, prior to the pulse-response measurement the above mentioned 500 Hz square wave was applied to the driver and the tip–substrate distance was regulated with the amplitude of the 500 Hz component of the cantilever deflection being detected with a lock-in amplifier. By setting the feedback reference to 90% of the full amplitude, the cantilever proved to remain stably at about 1 nm from the substrate. After the distance was stabilized the feedback loop was held and the duty cycle of the input signal was immediately changed to 99.5% so that a square pulse with a duration of 10 μs was applied to the driver circuit. Since a soft cantilever with a nominal spring constant of 0.03 N/m was used, once it drifted into contact with the substrate during data acquisition, it could hardly be separated unless the sample stage was moved. Thus, such data could be readily discriminated after the acquisition and be excluded from analysis.

**Figure 5:**
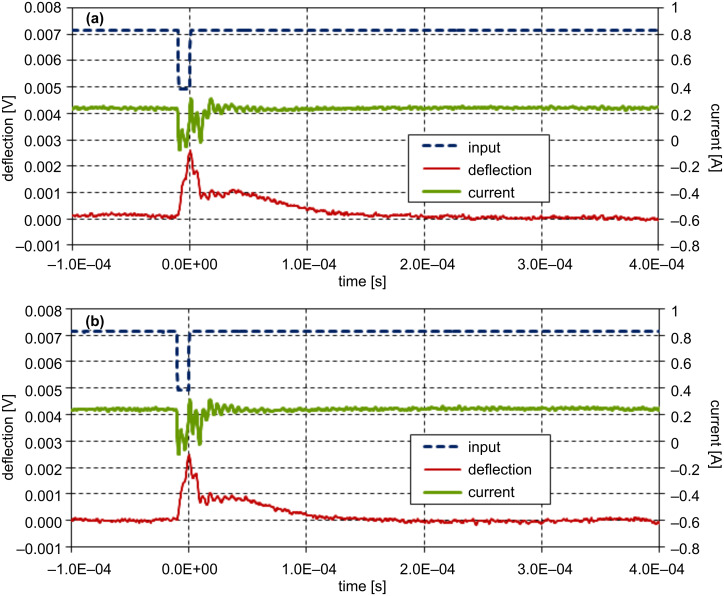
Comparison of pulse responses recorded at 300 nm from the substrate (a) and in close proximity (b). The fine broken, thick solid, and fine solid lines indicate the input-voltage signal, current in the electromagnet, and cantilever deflection, respectively. The cantilever swings toward the substrate with an amplitude of about 1 nm.

Figure 5a shows the input pulse signal, the current, and the cantilever deflection at a tip–substrate gap of ca. 300 nm, and Figure 5b the same signals with the tip brought close to the point of contact. Since the feedback of the sample stage is temporarily held during the pulse measurement, the accurate value of the tip–substrate gap is not known for the data shown in Figure 5b. The cantilever deflection signal swings by 2.5 mV, which corresponds to a downward deflection of about 1 nm, and then relaxes to the rest position in both Figure 5a and Figure 5b. Although these two response waveforms seem alike at a glance, a closer look reveals that the one recorded in proximity to the substrate decays faster. The waveform segments corresponding to a time section of 0 to 0.3 ms in Figure 5a and Figure 5b were extracted for analysis.

**Figure 6:**
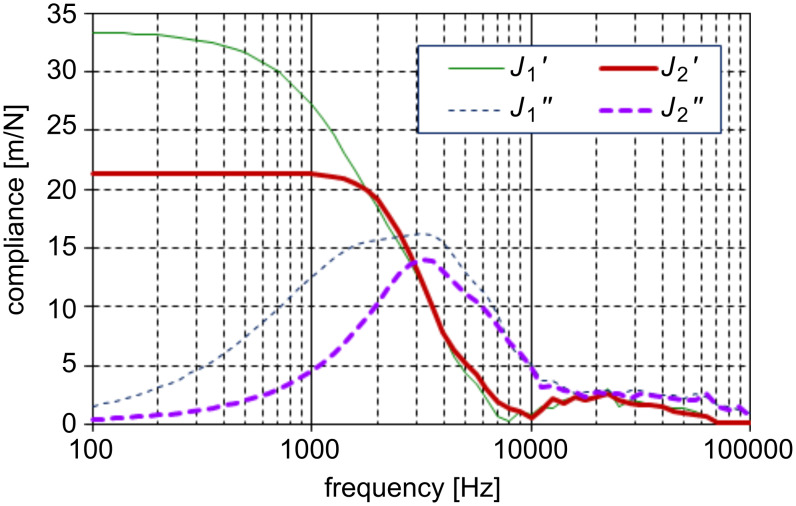
Complex compliance of cantilever–water system calculated by Fourier–Laplace transformation of the response signals in Figure 5(a) and Figure 5(b). The solid and broken lines indicate the real and imaginary parts, respectively, and the fine and thick lines the data at 300 nm and in close proximity, respectively.

**Figure 7:**
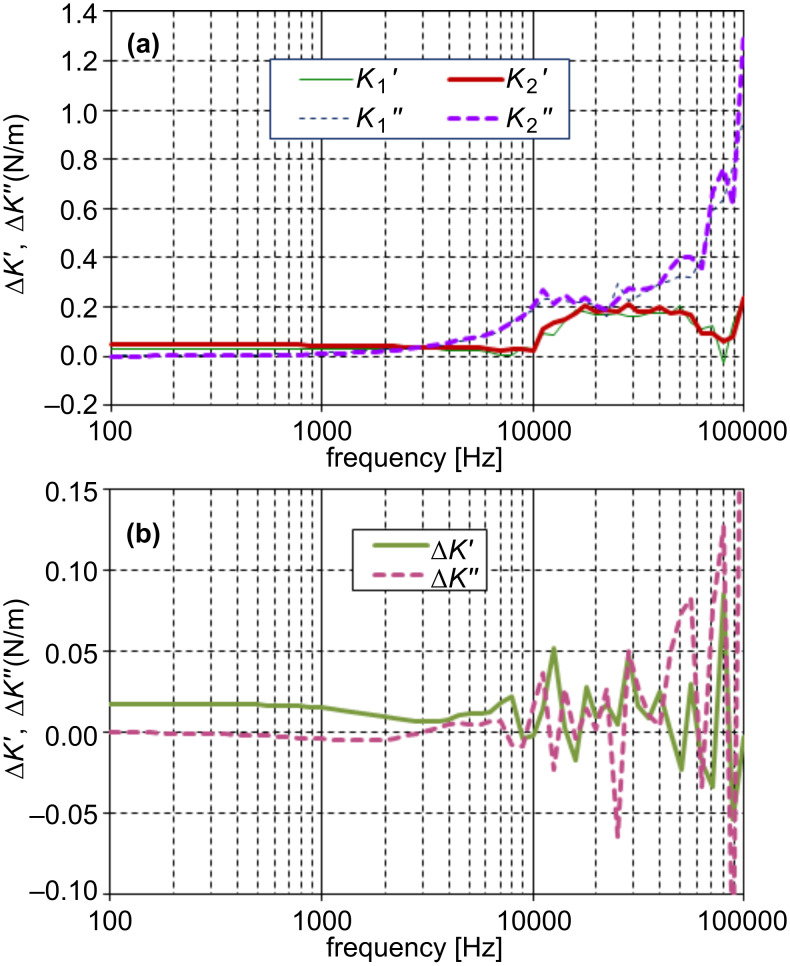
Derivation of the viscoelasticity of hydrated water. The compliance data shown in Figure 6 are inverted to give the elasticity (a). Solid and broken lines indicate the real and imaginary parts, respectively, and fine and thick lines the data at 300 nm and in close proximity, respectively. Subtraction of the two data sets gives the real (solid line) and imaginary (broken line) parts of the viscoelasticity (b).

Figure 6 shows complex compliances


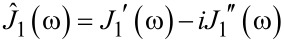


and


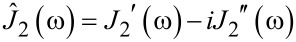


derived by Fourier–Laplace transformation of the response waveforms shown in Figure 5a and Figure 5b, respectively. One common and pronounced feature in these compliances is a peak at about 3 kHz in the imaginary parts and the corresponding drop in the real parts. These features are typical of a relaxation determined by a single pair of simple elastic and viscous elements, and is immediately attributed to the cantilever response. For further analysis the compliances 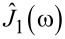 and 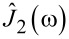 were inverted to complex elasticities


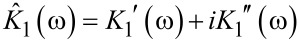


and


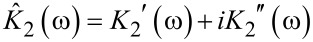


as,

[2]
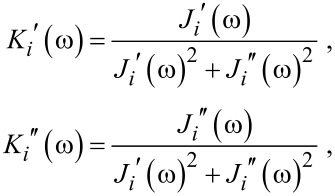


where *i* = 1,2, as shown in Figure 7a. Since elasticities of parallel mechanical elements are additive, the stiffness of the hydrated water interacting with the probe 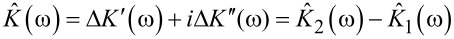 can be derived as shown in Figure 7b. The above mentioned response of the cantilever is well suppressed by subtraction, with the exception of a slight subtraction error, especially evident as a negative value of 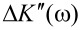 around 1 kHz. Since the original response signal in Figure 5 decays in about 0.3 ms, the elasticity data below 3 kHz is not so informative. Probing a frequency regime below this would require a cantilever having a longer relaxation time in water. Therefore the positive constant value of 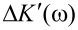 in the low-frequency regime in Figure 7b is not realistic. This is obvious also from the fact that a fluid cannot maintain finite stiffness down to zero frequency unless it is completely solidified. However, this positive value is maintained in the higher frequency regime, and this stiffening accounts for the observed shortening of the relaxation time. On the other hand, 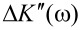 seems to start increasing above 10 kHz, although it substantially perturbed by noise. Similar behaviors of 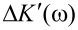 and 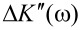 were observed in a different data acquired in the same experimental run, apart from a irreproducible singularity around 6 kHz attributed to the influence of the residual 1st mode resonance of the cantilever. A constant value of 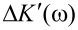 hints at a system having only a single relaxation time. Then, however, it should show a simple linear increase in 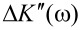, which seems contrary to the data in Figure 7b. It is probable that the apparent onset in 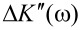 at 10 kHz is actually the point at which the signal reaches a measurable level. For a decisive conclusion a more refined measurement is indispensable.

In the previous report on step-response measurement, the response signal in the cantilever deflection exhibited a substantially longer decay time in the proximity of a mica substrate in water [25]. In the present experiment the result seems quite the contrary, that is, the decay time seems to be rather shortened by hydration, as shown in Figure 5. Although the properties probed by the step-response and pulse-response measurements are basically similar for an elastic sample, these two methods may lead to different outcomes for quasi-fluid samples. A quasi-fluid sample loaded with a step stress continues to relax until it reaches a new equilibrium state. This effect leads to a lateral flow of the fluid and is detected as a long decay time of the cantilever position and hence a large apparent drag coefficient [31]. This lateral flow is considered to cause coupling between the longitudinal response of the hydration layer and its shear property, and thus complicates the data analysis [19]. In the case of the pulse-response measurement, the effect of such a flow is expected to be weaker.

In the present measurement the feedback loop for regulation of the tip–sample gap must be suspended during the period of data acquisition. There is no insurance against fluctuation of the true tip–sample distance due to thermal or mechanical drift, although it was confirmed after data acquisition that the tip had not drifted into contact with the substrate. For further progress it is necessary to combine the present method with operation modes having resolution along the substrate-normal direction, such as the force-profile measurement. It should also be noted that the properties of solvation layers are strongly dependent on the number of layers. In order for the probe not to destroy the layer with the pulse motion, the S/N ratio must be improved such that a pulse with smaller amplitude is sufficient. Since hydration on mica is considered to become distinguished when the film thickness is reduced to several molecular layers, i.e., less than 1 nm, an improved measurement with a smaller pulse magnitude is expected to reveal more detail on the properties of the hydration.
